# Ticks and Tick-Borne Diseases of Livestock in the Middle East and North Africa: A Review

**DOI:** 10.3390/insects12010083

**Published:** 2021-01-19

**Authors:** Nighat Perveen, Sabir Bin Muzaffar, Mohammad Ali Al-Deeb

**Affiliations:** Department of Biology, United Arab Emirates University, Al-Ain 15551, UAE; 201790740@uaeu.ac.ae (N.P.); s_muzaffar@uaeu.ac.ae (S.B.M.)

**Keywords:** Arab countries, tick distribution, tick fauna, tick-borne diseases, pathogens

## Abstract

**Simple Summary:**

The Middle East and North Africa represent a hyper arid region of the world. Humans in these regions have historically kept domestic livestock usually under harsh environmental circumstances. With recent human development, especially in the Middle East, the livestock industry has increased. Livestock is affected by ticks and tick-borne diseases on which there has been relatively few studies in this region. This review paper aims to (i) examine the diversity of ticks infesting livestock, (ii) assess the variety of pathogens in animals and humans, and (iii) to identify gaps in our understanding of tick biology and tick-borne disease transmission in the region. We found 55 tick species on livestock including camels, cows, goats and sheep, belonging to eight genera. Fifteen tick-borne pathogens were reported from livestock in the region. We highlight the magnitude of the tick problem in the region and evaluate the control efforts currently in place. We suggest that disease control and prevention could be achieved effectively through collaborative work among all stakeholders such as funding international research projects and establishing joint control programs to combat cross-border movement of ticks.

**Abstract:**

Ticks are important vectors of an array of viral, bacterial and protozoan pathogens resulting in a wide range of animal and human diseases. There is limited information in the literature about tick species in the Middle East and North Africa (MENA) countries, even though they have suitable climate and vegetation for ticks and their hosts. We reviewed the occurrence of tick species and the pathogens they transmit from the MENA on published papers from 1901–2020. We found taxonomic records of 55 tick species infesting livestock representing the following eight genera: *Ornithodoros*, *Otobius*, *Amblyomma, Dermacentor, Haemaphysalis, Hyalomma, Ixodes,* and *Rhipicephalus*. In addition, 15 pathogens were recorded causing diseases of significance, with Crimean–Congo hemorrhagic fever, theileriosis, babesiosis and anaplasmosis being widely distributed diseases in the region. In recent decades, there has been increasing trends in disease occurrence and movement associated with global movement of humans and global trade of animals. We suggest that disease control and prevention could be achieved effectively through good integration between public health, veterinary medicine and animal management, and ecological approaches. We recommend further research in the areas of tick ecology and tick born-disease transmission. Furthermore, we suggest evaluation and improvement of disease control policies in the region.

## 1. Introduction

Ticks play a major role in transmitting infectious diseases. Emerging or re-emerging infectious diseases are important global problems of great concern to humans as well as to animal health, with many pathogens being able to infect multiple species [1,2,3,4]. With increasing antimicrobial resistance among bacterial pathogens, there has been an increase in the occurrence of zoonotic diseases, sometimes causing widespread outbreaks with considerable domestic animal, wildlife and human morbidity and mortality [4]. Therefore, infectious diseases have been recognized as an increasing threat to the general public and animal husbandry. Accelerating climate change carries serious threats for public health and society. Global warming and the unstable climate are playing an ever-increasing role in driving the global emergence, resurgence and redistribution of infectious diseases [5]. Globalization and climatic abnormalities have allowed parasites to invade into new geographic areas or establishing ranges in common localities, giving rise to epidemics and epizootics worldwide [1,2,3,6].

The Arabian Peninsula is globally important as a source of energy, with vast oil and gas reserves that supply the energy demands for the entire world. Consequently, this area went through a vast development and changes. One of the side effects of this development has been the expansion of farming, particularly in the Arabian Peninsula, primarily to support the demands of the rising population [7]. Camels, cows, buffaloes, sheep and goats are farmed using a combination of traditional and modern farming techniques [8]. These animals produce considerable amounts of milk, meat, wool and hides [9] and ticks constitute a major threat to the livestock industries in the MENA (Middle East and North Africa). Ticks have great significance from economic, veterinary, and human health perspectives because of their capacity to transmit a variety of diseases [10]. The ixodid tick genus *Hyalomma* alone causes enormous losses to the products arising from camels and other livestock across MENA region [11,12,13]. The roles of diverse assemblages of ticks as vectors and reservoirs of pathogens and their impacts on livestock are not well understood or characterized, although many ticks and tick-borne diseases are known from the Arab region.

The aim of this review is to determine the tick-borne diseases and tick fauna prevailing in the Arab countries. A clear understanding of the abundance of tick species, their role as reservoirs and vectors of pathogens, as well as their geographical distribution, is pivotal in assessing the threat of disease outbreaks and controlling tick-borne diseases in the future.

The objectives of our study were to:

- Review the diversity of ticks of livestock in the MENA region;
- Review the variety of pathogens transmitted by ticks, with especial emphasis on emerging or re-emerging tick-borne diseases;
- Identify gaps in the science of tick biology and tick-borne disease transmission in the region and provide recommendations on the management of ticks and tick-borne diseases in the region.

## 2. Methodology

### 2.1. Data and References Collection

We used Google Scholar, Science Direct, Scopus, Web of science and PubMed to collect the available research papers about ticks, tick-borne diseases and pathogens in Arab countries. We also searched for resources like reports that include tick species and tick-borne diseases in MENA countries. Two hundred and ninty four references have been used in this review paper. For each Arab country, we searched relevant literature by using the following keywords: ‘the name of the country’, ‘ticks’, ‘tick species’, ‘tick-borne diseases’, ‘tick-borne pathogens’, ‘tick distribution’, and ‘livestock’. The list of the ticks presented here includes almost all species on livestock from Arab world reported in published papers and books.

### 2.2. Tick Nomenclature

For nomenclature, we presented tick species names according to Guglielmone et al. [14]. In this paper, all the old tick names are replaced by the names presented in the aforementioned checklist. For example, the genus *Boophilus* is replaced by *Rhipicephalus* and the species *Hyalomma detritum* is replaced by *Hyalomma scupense, Hyalomma erythraeum* is replaced by *Hyalomma impeltatum.*

## 3. Geography of Arab Countries

**Figure 1:**
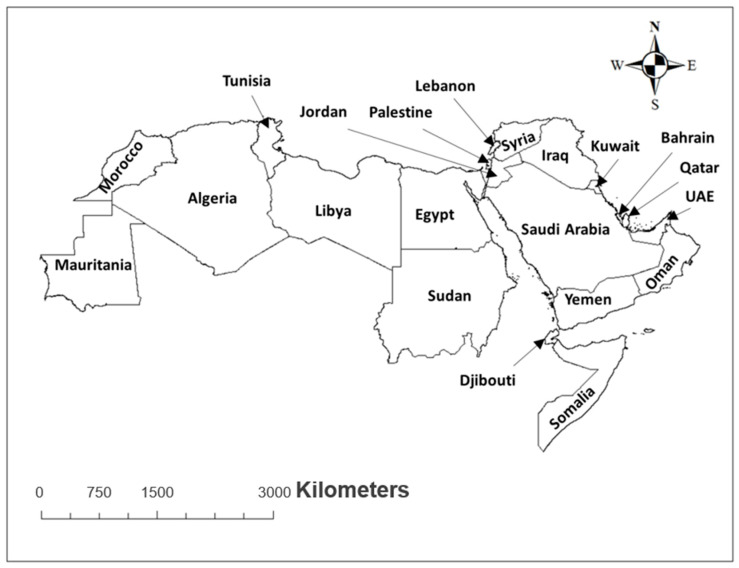
Regional map showing Arab countries.

The Arab World consists of 22 countries in the MENA region: Algeria, Bahrain, Comoros, Djibouti, Egypt, Iraq, Jordan, Kuwait, Lebanon, Libya, Mauritania, Morocco, Oman, Palestine, Qatar, Saudi Arabia, Somalia, Sudan, Syria, Tunisia, United Arab Emirates, and Yemen [15] (Figure 1). These states occupy an area extending from the Atlantic Ocean to the Zagros Mountains in southwest Asia [16]. The overall total population of the Arab nations in the world is estimated to be approximately 427 million [17].

The Arab countries occupy 14,291,469 km^2^, which comprises approximately 10.2% of the world’s land mass. Out of this, 72.45% is located in Africa and 27.55% is located in Asia. This region comprises of two distinct parts, eastern and western. Generally, the Asian part of the Arab World is the Arab East while the African part is the Arab West [18,19].

Ninety percent of the region consists of arid, semi-arid and dry sub-humid areas. The area is characterized by harsh environment, limited water resources and arable lands. Throughout its long history these lands were the main source of grain and animal production [16,18].

## 4. Biogeography of Ticks

About 80% of the world’s cattle are infested with ticks. As a result, ticks are economically important ectoparasites of livestock. Ticks have negative effect on their vertebrate hosts due to blood feeding causing irritation, allergic dermal reactions, severe paralysis and tick toxicosis [20,21]. The economic loss due to tick-borne diseases among ruminants in tropical and subtropical areas is calculated to be several billion dollars annually [22,23]. Generally, the climate of the Arabian Peninsula is characterized by hot dry summers and mild winters with periods of high relative humidity (especially in the coasts) [24]. North Africa, in comparison has a more Mediterranean influence in its climate, although the central portions have arid deserts. Ixodid ticks are distributed in areas where they have sufficient animal hosts to sustain their complex life cycles. Hard ticks with two-host or three-host life cycles must occur in distributions that overlap with all their hosts (assuming that different instars feed on different host species). In the case of livestock, ticks could potentially use a combination of livestock species to complete their life cycles. In some cases, species such as wild rodents or insectivores (such as hedgehogs) could be used at least for the earlier stages (larvae and nymphs). These species are especially important in the traditional farming systems (such as *izbas* or local farm enclosures), where livestock holding areas are accessed by small mammals [25,26].

## 5. Diversity and Distribution of Ticks in Arab Countries in Middle East and North Africa

### 5.1. Tick Species Diversity in MENA Region

A total of 55 species of ticks in eight genera have been documented from livestock in North Africa and the Arabian Peninsula (Table 1). The soft ticks (Argasidae) are represented by two genera, *Ornithodoros* and *Otobius*. *Ornithodoros savignyi* appears to be the most widespread, being recorded from most of the Middle East and into parts of North Africa. The hard ticks (Ixodidae) are represented by six genera (*Amblyomma*, *Dermacentor*, *Haemaphysalis*, *Hyalomma, Ixodes* and *Rhipicephalus*) of which *H. impeltatum*, *H. dromedarii*, *R. annulatus* and *R. sanguineus* are widely distributed in the MENA region. Among these, *Hyalomma dromedarii* is the most common tick with high prevalence due to large-scale camel farming.

Arab countries have a suitable habitat for ticks, however, taxonomic studies on tick species in most countries are very limited. The majority of taxonomic studies on ticks of the Arab world were generally focused on hard ticks. The maximum numbers of tick species from livestock are documented in Egypt, Sudan and Jordan (Table 1). *Hyalomma* and *Rhipicephalus* are the most common genera reported from livestock in almost all Arab countries. *Hyalomma* genus serves as the vector and reservoir of CCHF virus [27]. In several countries, we found a small number of published papers on ticks’ species and tick-borne pathogens. This indicated the presence of a gap in the research dedicated to tick and tick-borne diseases. There is also need to look into the various aspects of tick distribution to manage future emerging and re-emerging tick-borne diseases and disease movement especially Arab countries which are sharing borders with each other.

### 5.2. Tick Distribution by Country

#### 5.2.1. Algeria

Ticks are well-known to transmit pathogens which threaten the health and welfare of companion animals and man in Algeria. In 1923, *O. savignyi* was reported in Wargla as a parasite of camels [32]. In recent years, the scope and importance of ticks and emergent tick-borne diseases have been increased dramatically in Algeria and 16 tick species have been reported from livestock. During 1981–1983, ticks were collected for two years from cattle in Algeria and separated into six genera and twelve species including *R. bursa, R. turanicus, R. sanguineus, R. annulatus, I. ricinus, H. punctata, D. marginatus, H. scupense, H. impeltatum, H. marginatum, H. excavatum and H. lusitanicum* [49]. Same kind of study was conducted in Northern Algeria between May 2001 and November 2003 on various mammals and ticks were recognized at species level as *R. sanguineus, H. marginatum, H. scupense, R. bursa* and *R. turanicus* [59]. However, *I. ricinus* ticks were collected on cattle and identified, in North-eastern Algeria from December 2005 to March 2006 [60]. Djerbouh et al. [53] determined tick species including *H. dromedarii, H. rufipes, H. impeltatum,* and *H. impressum* from camels in four regions of Southern Algeria. Whereas 11 species of ticks were recorded on domestic animals including *H. punctata, D. marginatus, R. sanguineus, R. bursa, H. lusitanicum, H. scupense, H. excavatum, H. marginatum and I. ricinus* during a study conducted in 2012, 2013 in Algeria [56]. Five genera including *Hyalomma, Rhipicephalus, Dermacentor, Ixodes, Haemaphysalis* and sixteen species consist of *O. savignyi, R. bursa, R. turanicus, R. sanguineus, R. annulatus, I. ricinus, H. punctata, D. marginatus, H. dromedarii, H. rufipes, H. impressum, H. scupense, H. impeltatum, H. marginatum, H. excavatum* and *H. lusitanicum* have been reported in Algeria from livestock according to our review (Table 1).

#### 5.2.2. Egypt

Ticks, their feeding habits and other disease causing aspects have been reported in early historical time from Egypt [70]. Over a number of years studies have been carried out in Egypt on ticks of migratory birds and wildlife that may transmit pathogens of man and animals [37,71]. Various tick species from Egypt have been reported to infest camels, cows, buffaloes, and sheep [62,72]. *O. savignyi* was described in a report on African Ixodoidae as an Egyptian tick specimen [73]. The genera *Hyalomma*, and *Rhipicephalus* comprise the most important ixodid ticks infesting animals, specifically *H. excavatum, H. dromedarii, H. impeltatum, H. marginatum, R. annulatus,* and *R. sanguineus* [62]. The cattle tick, *R. annulatus*, is considered to be the most important economic pest infesting cows in Egypt. *H. dromedarii, H. impeltatum, H. excavatum, H. anatolicum, H. truncatum, H. marginatum, H. rufipes, H. turanicum, R. annulatus, R. sanguineus, R. turanicus, R. guilhoni, R. camicasi, A. lepidum, A. marmoreum,* and *A. variegatum,* were collected from different localities in Egypt. *Hyalomma* species were found on camels and cows. *R. annulatus* was found only on cows, *Rhipicephalus* species found on camels and sheep, *Amblyomma* species were found on imported camels [41]. For detection of piroplasmids such as *Babesia* and *Theileria*, ticks were collected from sheep, goats, cattle and camels during 2001 and identified as *R. appendiculatus, R. bursa, R. turanicus* and *H. parva* on sheep, *H. excavatum* and *H. sulcata* on goats, *H. lusitanicum* on cattle and *H. dromedarii*, *H. impeltatum, H. marginatum* and *H. anatolicum* on camels. Two pathogens *B. ovis* and *T. ovis* were detected in livestock [74]. Whereas in another study *H. dromedarii, A. lepidum, A. variegatum,* and *R. pulchellus* were found on camels and *T. annulata* was detected in *H. dromedarii* [43]. Asmaa et al. [51] conducted a study to determine the associated causes of tick infestations in ruminants and reported that the *R. annulatus* is the most dominant tick species affecting livestock followed by *H. anatolicum* and *R. turanicus*. In a similar study from July to October 2008, Abdel-Shafy et al. found that cows were infested with *H. excavatum* and *R. annulatus* and camels were infested with *H. dromedarii*, *H. impeltatum*, and *H. marginatum* [54]. Furthermore, a study was carried out during the period from December 2014 to November 2015. A total of 1540 ticks were collected, four ixodid tick species; *H. dromedarii, A. lepidum, A. variegatum,* and *R. pulchellus* were found on camels [43]. So far, twenty-two tick species from four genera have been recorded on livestock in Egypt including *H. excavatum, H. dromedarii, H. impeltatum, H. marginatum, H. anatolicum, H. truncatum, H. rufipes, H. lusitanicum, H. turanicum, H. sulcata, H. parva, R. appendiculatus, R. bursa, R. annulatus, R. sanguineus, R. turanicus, R. guilhoni, R. camicasi, R. pulchellus, A. lepidum, A. marmoreum,* and *A. variegatum* (Table 1).

#### 5.2.3. Iraq

Ticks and tick-borne diseases constitute a major challenge for livestock health and production in Iraq [75]. Iraq has a huge population of small ruminants estimated at 10 million heads in 2018 [76], and these animals produce considerable amounts of milk, meat, wool, skin and contribute in Iraq’s economy. Hoogstraal and Kaiser [77] listed 21 species from several genera including *Argas*, *Ornithodoros*, *Haemaphysalis*, *Hyalomma*, *Ixodes* and *Rhipicephalus*. Ticks of domestic animals are relatively well documented in Iraq [46,52,75,77,78,79,80,81,82,83,84,85,86,87,88,89,90,91,92,93,94]. Sheep and goats are main domestic animals and play an integral in the agricultural economy. They are raised in large numbers throughout the country for their meat, milk and wool [79] and are subject of tick infections with both ixodid and argasid ticks [80]. Twelve tick species, namely *R. kohlsi, R. annulatus, R. leporis, R. turanicus, R. bursa, O. erraticus, H. erinacei, H. sulcata, H. scupense, H. turanicum,* and *H. anatolicum* were reported [46]. A study on hard ticks affecting cattle, sheep, and goats indicated that cattle were infested with *H. marginatum* and *H. anatolicum* while *R. bursa, R. turanicus, H. parva,* and *Hyalomma* spp. were reported from sheep and goats. The peaks of infestation occurred in the middle of June until end of July [47]. However, in another study, the high infestation was reported in May and July months, respectively. Two genera (*Hyalomma* and *Rhipicephalus*) were recorded from 521 collected ticks from sheep and cattle, and 13 species were identified; *H. anatolicum*, *H. excavatum, H. asiaticum*, *H. marginatum, H. turanicum, H. scupense, H. impeltatum, R. turanicus*, *R. bursa, R. sanguineus, R. annulatus*, *R. kohlsi*, and *Hyalomma* spp. The most encountered tick species was *H. anatolicum* in *Hyalomma* genus, *R. turanicus* in *Rhipicephalus* genus [52]. The literature review revealed that *Rhipicephalus, Hyalomma* and *Haemaphysalis* are the main genera infesting livestock in Iraq [8,46,52,79,81,82,84,86,87,88,89,90,95,96,97] (Table 1).

#### 5.2.4. Jordan

Few studies have been carried out on tick fauna [48,98,99] and the Jordanian ticks are poorly studied as compared to neighboring countries [47,100,101,102]. Based on the collection made during 1952–1954, Hoogstraal and Kaiser [40] reported on the tick fauna. That collection contained several species of both ixodid and argasid ticks. They found *R. kohlsi* infesting sheep and goats. To define the tick fauna associated with domestic ungulate animals, El-Rabie et al. [48] collected fourteen species and subspecies of ixodid ticks including *H. parva, H. sulcata, R. sanguineus, R. bursa, R. kohlsi, R. annulatus, H. anatolicum, H. excavatum, H. marginatum, H. turanicum, H. scupense, H. impeltatum, H. dromedarii,* and *H. schulzei*, representing three genera (*Hyalomma, Rhipicephalus, and Haemaphysalis*) from native sheep, goats, camels and cattle. *Haemaphysalis parva* was the most common species among the 9545 specimens examined. Although Saliba et al. [28] reported argasid including *Argas* sp., *Ornithodoros coniceps, O. erraticus, Ornithodoros tholozani, O. salahi, O. lahorensis* and 17 ixodid species comprising of *H. marginatum, H. rhipicephaloides, H. anatolicum, H. dromedarii, H. impeltatum, H. schulzei, H. scupense; R. sanguineus, R. turanicus, R. camicasi, R. bursa, H. erinacei, H. sulcata, H. parva, R. annulatus, R. kohlsi,* and *Ixodes* sp. from East Jordan and the West Bank. No major attention was given to the tick’s role in transmission of tick-borne pathogens. Twenty nine tick species from six genera including *Haemaphysalis, Hyalomma, Rhipicephalus, Ixodes, Ornithodoros* and *Argas* have been reported in Jordan. However, we have not included ticks from genus *Argas* in Table 1, because in Arab countries almost all ticks belong to this genus reported from birds/wildlife.

#### 5.2.5. Kuwait

Very little work has been carried out on ticks infesting ruminants. Converse and Moussa [103] collected ticks in May–June in 1973 in Kuwait, Iraq and Yemen and described the species as *H. dromedarii, H. impeltatum, H. schulzei.* Further, four tick species including *R. annulatus, H. anatolicum, H. marginatum,* and *R. sanguineus* have been reported in 1999 in Kuwait in Disease Vector Ecology Profiles (DVEPs) that summarize unclassified literature on medically important arthropods, vertebrates and plants that may adversely affect in regions around the world [29]. Seven tick species including *R. annulatus, H. dromedarii, H. impeltatum, H. schulzei, H. anatolicum, H. marginatum,* and *R. sanguineus* have been documented from Kuwait (Table 1).

#### 5.2.6. Lebanon

Limited data is available about tick presence in domestic ruminants in Lebanon. Six tick species have been reported to be endemic in Lebanon: *R. annulatus, R. sanguineus*, *H. anatolicum, H. excavatum H. schulzei*, and *D. marginatus* [104,105,106]. Lately, research was conducted with the aim of providing an analysis of tick presence and distribution. The ticks were randomly collected from domestic ruminants (cattle, sheep, and goats) at 37 farms, in six provinces between June and September 2014. Ticks belonged to four Ixodidae genera: *Rhipicephalus, Haemaphysalis, Dermacentor* and *Hyalomma* and seven species: *R. annulatus*, *R. turanicus*, *R. sanguineus, R. bursa, H. anatolicum*, *H. punctata* and *D. marginatus*. *R. turanicus* and *H. anatolicum* were found on cattle, sheep, and goats, *R. annulatus* on cattle and sheep, *R. sanguineus, D. marginatus* and *H. punctata* on sheep and goats, while *R. bursa* was collected only from sheep. Some of the identified species were recorded for the first time [45]. However, in 2018, only five tick species were recorded as *H. anatolicum, R. annulatus, R. bursa, R. sanguineus* sensu lato, and *R. turanicus* from farm ruminants. Spotted fever group *Rickettsiae* were molecularly identified and characterized in these ticks [107]. Recently, three tick species (*H. punctata, H. parva, D. marginatus*) are identified from human hosts [108]. In Lebanon, ten tick species comprising *D. marginatus, H. anatolicum, H. schulzei, H. excavatum, H. parva, H. punctata, R. bursa, R. annulatus, R. sanguineus,* and *R. turanicus* have been documented in the literature (Table 1).

#### 5.2.7. Libya

A number of studies on animal parasitology in Libya were carried out before World War II by Italian workers [109] and later by Hoogstraal and Kaiser [35]. Italian authors were mainly interested in tick-borne human diseases and reported *O. foleyi* (vector of tick bite fever) [110], *O. tholozani* (vector of relapsing fever) [111] and *R. sanguineus* (vector of boutonneuse fever) [112]. Hoogstraal and Kaiser [35] provided details concerning 14 species of ticks from Libya. However, there are considerable gaps in tick research in the country. In 1991, Beesley and Gabaj [113] identified *R. bursa*, *R. microplus*, and *R. decoloratus* during 4 years survey in Libya on cattle, goats, sheep and camels. Though, *R. bursa* was originally reported from Libya [114], this identification was considered incorrect [115]. In 1992, thirteen species of ixodid ticks and two species of argasid ticks were documented from farms in Libya such as *R. annulatus*, *R. microplus*, *R. decoloratus*, *R. sanguineus*, *R. evertsi*, *R. bursa*, *H. anatolicum*, *H. excavatum H. dromedarii*, *H. franchinii*, *H. impeltatum*, *H. rufipes*, *H. turanicum*, and *O. foleyi*. *Hyalomma, Rhipicephalus, Argas* and *Ornithodoros* were the main genera. *H. dromedarii* was found abundant on camels, *H. impeltatum* on sheep and *H. excavatum* on cattle. *H. dromedarii* was the most abundant tick found overall [30]. Fifteen species have been documented in Libya including *R. annulatus*, *R. microplus*, *R. decoloratus*, *R. sanguineus*, *R. evertsi*, *R. bursa*, *H. anatolicum*, *H. excavatum H. dromedarii*, *H. franchinii*, *H. impeltatum*, *H. rufipes*, *H. turanicum*, *O. tholozani* and *O. foleyi* (Table 1).

#### 5.2.8. Mauritania

In Mauritania, a demographic transition occurred between the 1970s and 2000s, characterized by massive rural-to-urban migration. Nomadic habits, such as possessing domestic animals, were maintained. In cities with a high population density of humans, especially in areas where zoonoses are prevalent, this practice represents a major risk for human populations [65]. In 2003, 38 persons were infested with Crimean–Congo hemorrhagic fever (CCHF) virus in Mauritania [65]. A study was conducted to investigate the magnitude and conditions of emergence of this first urban CCHF outbreak in Mauritania. Ticks collected from animals in livestock markets represented two genera (*Hyalomma* and *Rhipicephalus*) and six species of ticks (*H. dromedarii, H. impeltatum, H. rufipes*, *Hyalomma* sp., *R. sanguineus* and *R. evertsi*). Members of the genus *Hyalomma*, the principal vector of CCHF virus, were found in the same proportion as genus *Rhipicephalus* [65]. Subsequently, eight tick species were reported to be infested with CCHFV (*A. variegatum, R. decoloratus, R. geigyi, H. dromedarii, H. impeltatum, H. impressum, H. rufipes, H. truncatum,* and *R. guilhoni* [57,65]. In 2008, Sylla et al. reported two soft ticks *Ornithodoros sonrai* and *O. savignyi*, and seven hard ticks *H. excavatum, H. dromedarii, H. impeltatum, H. impressum, H. rufipes, H. nitidum* and *H. truncatum* to document the climate change effects and distribution of ticks (Acari: Ixodida) in Senegal and Mauritania [36]. Fifteen tick species have been reported from Mauritania, for example *H. dromedarii, H. impeltatum, H. excavatum, H. rufipes, R. sanguineus, R. evertsi, A. variegatum, R. decoloratus, R. geigyi, H. impressum, H. nitidum, H. truncatum, R. guilhoni, O. sonrai and O. savignyi* (Table 1).

#### 5.2.9. Oman

Oman has relatively high diversity of flora and fauna especially certain arthropod parasites involved in disease transmission [116]. Ticks and their associated hosts have been reported in Oman since 1980 [117]. Papadopoulos et al. reported the following tick species from Oman: *O. foleyi, O. sonrai, Haemaphysalis indica, H. anatolicum, H. dromedarii, H. impeltatum, H. marginatum, H. turanicum, H. arabica* and *R. sanguineus* [118]. Further work revealed *A. variegatum, H. anatolicum, H. dromedarii, H. rufipes and Rhipicephalus* spp. from cattle, camels and goats [119]. Twelve tick species have been reported from Oman including *A. variegatum*, *R. annulatus*, *H. anatolicum*, *H. dromedarii*, *H. impeltatum*, *H. rufipes*, *R. sanguineus*, *O. foleyi*, *O. savignyi*, *H. indica*, *I. hoogstraali* and *H. turanicus* [29] (Table 1).

#### 5.2.10. Qatar

Tick fauna and tick-borne diseases are poorly studied in Qatar. Two species of ticks, *H. dromedarii* and *H. impeltatum*, have been reported on camels from Qatar [55,58] (Table 1).

#### 5.2.11. Saudi Arabia

The tick fauna associated with domestic animals is relatively well known in the Saudi Arabia [25,26,100,120]. Various tick species are indigenous to Saudi Arabia [25,26,121,122,123]. Hoogstraal et al. [100] documented 37 tick species and sub-species infesting livestock and wild animals and recognized various tick-host relationships. In a study of two consecutive years 1990 and 1991, and 8772 ticks were collected from livestock all across the Saudi Arabia, including one argasid (*A. persicus*) and 15 ixodid species (*A. variegatum, A. gemma, R. kohlsi, H. sulcata, H. anatolicum, H. excavatum, H. arabicum, H. dromedarii, H. impeltatum, H. rufipes, H. turanicum, H. schulzei, Rhipicephalus camicasi, R. praetextatus,* and *R. turanicus*) [67]. *H. dromedarii* is the most prevalent species in Saudi Arabia [124]. Al-Shammery et al. conducted a study to find differentiation between common tick species using molecular biology techniques and identified *H. dromedarii* and *R. annulatus*. Both tick species were collected from infested camels and cattle in the animals resting house at Hail region [125]. Literature review revealed that *Amblyomma*, *Haemaphysalis, Hyalomma* and *Rhipicephalus* are the major genera comprising of seventeen species infesting livestock in Saudi Arabia (Table 1).

#### 5.2.12. Sudan

Sudan includes tropical forest, various types of forested and un-forested grasslands, semi-desert, and extreme desert. A large proportion of the ticks of tropical Africa occurs in Sudan. Among the large numbers of specimens studied from a wide variety of hosts, 63 tick species were listed infesting variety of domestic and wild animals including reptiles and birds [126]. Whereas Karrar et al. [127] mentioned 16 ticks species in their study of ecology and host-relationships of ticks infesting domestic animals in Kassala Province. Jongejan et al. [128] identified twenty-four adult ixodid tick species, infesting livestock and some wildlife hosts along the Blue and White Nile. More than 70 species of ticks are identified from Sudanese tick fauna, many of which are proven vector of major tick-borne diseases [129,130,131]. Three genera (*Hyalomma, Amblyomma,* and *Rhipicephalus*) and various species of ticks were identified from cattle, sheep, goat and camels. These include *A. lepidum, A. variegatum, H. anatolicum, H. dromedarii, H. impeltatum, H. rufipes, H. impressum, H. truncatum, H. scupense, R. annulatus, R. decoloratus, R. evertsi, R. guilhoni, R. muhsamae, R. praetextatus, R. appendiculatus, R. simus* and *R. sanguineus* [64,66,69,132]. Recently, four genera and fourteen species of ticks reported on cattle. These were *R. evertsi, R. decoloratus, R. sanguineus, R. turanicus, R. camicasi, R. annulatus, H. dromedarii, H. impeltatum, H. rufipes, H. anatolicum, H. truncatum, H. excavatum, A. variegatum,* and *A. lepidum* [63,133]. Overall, 22 species belonging to four genera have been documented from livestock in Sudan through literature (Table 1).

#### 5.2.13. Syria

The livestock sector has a significant impact on the Syria’s economy. The central region is ideal for livestock farming with ample grazing lands. Grazing dairy cattle in pasture may acquire the infection during tick infestation [134]. Six tick species have been reported in 1999 including *R. annulatus, H. anatolicum, H. excavatum, H. impeltatum, H. marginatum* and *R. sanguineus* [29] (Table 1).

#### 5.2.14. Tunisia

Little is known about tick species and tick-borne diseases in Tunisia. Fourteen tick species were reported from Tunisia in different time periods by different researchers. *H. scupense, H. marginatum, H. impeltatum, H. excavatum, H. rufipes, R. turanicus, R. sanguineus,* and *R. bursa* were reported from cattle, *H. dromedarii* from camels, *I. ricinus*, *H. punctata R. annulatus*, *H. sulcata, H. scupense* and *H. franchinii* ticks were documented from animals such as sheep, goats and cows (Table 1) [50,135,136,137,138,139,140,141].

#### 5.2.15. United Arab Emirates (UAE)

In the UAE, there are limited studies on the ticks and tick-borne diseases of domestic animals including camels, cattle, sheep and goats. However, ticks are reported round the year from camels in recent findings [142], which are continuous threat to livestock industry in UAE and people who are in close contact with these animals. Therefore, there could be more chances of emergence and re-emergence of tick-borne diseases. Various tick species including *H. anatolicum, H. excavatum, H. impeltatum, H. dromedarii, H. marginatum, H. truncatum, H. hussaini, R. appendiculatus, R. evertsi, R. pulchellus, R. sulcatus, A. gemma* and *A. lepidum* were identified from livestock and camels during an outbreak of Crimean–Congo hemorrhagic fever in the UAE in 1994–1995 [39]. In 2010–2011, a study was conducted for screening of tick-borne diseases (protozoan and bacterial diseases) in Al-Ain, UAE and camel tick was identified as *H. dromedarii* on molecular basis [143]. In addition, in 2010–2011, *H. dromedarii* was reported with high prevalence (98%) from UAE [144]. Recently, *H. scupense* and *H. rufipes* are reported from camels in annual report of Central Veterinary Research Laboratory in UAE [145]. Fifteen species have been recorded from livestock including camels in the UAE (Table 1).

#### 5.2.16. Yemen

The earliest report on ticks was prepared by Hoogstraal and Kaiser [40] who reported the results of a survey made in 1951; most of the species of ticks infested all types of domestic livestock and they included potential vectors of various important cattle diseases, notably theileriosis, babesiosis, anaplasmosis and heart-water. Pegram et al. [33] reported several tick species from camels, cattle, goats and sheep. Each tick species commonly infesting livestock recorded as transmitting viruses (and some also rickettsiae) causing human illness. Between 1300 and 2000 m in altitude, *A. variegatum* was more abundant on camels than on cattle and while cattle were infected with *R. annulatus.* Below 1500 m, *R. kohlsi* was more abundant on goats than on sheep and *H. sulcata* was found on sheep. Whereas *H. dromedarii* Koch was prevalent on livestock and *H. excavatum* was on camels. *H. anatolicum, H. impeltatum* and *H. rufipes, H. arabica, R. evertsi, R. simus, R. sanguineus* and *O. savignyi* ticks were also reported from various domestic animals. However in 1987, ten species of ticks were collected in the survey from the five hosts (cattle, sheep, goats, camels and donkeys): *A. variegatum, R. annulatus, R. kohlsi, H. excavatum, H. dromedarii, H. arabica, H. rufipes, R. evertsi, R. sanguineus,* and *R. simus* [146]. In another study, ticks of the group of *R. sanguineus* collected from camels, cattle, sheep and goats were examined. Adult ticks of three species in the group were identified: *R. camicasi, R. turanicus*, and *R. sanguineus s.s.* Host relationships and ecological affinities were also analyzed [68]. Nine species of ticks were reported in 1999 including *A. variegatum, R. annulatus, H. anatolicum, H. excavatum, H. dromedarii, H. impeltatum, H. rufipes, H. truncatum* and *R. sanguineus* (Anonymous, 1999). While Indigenous sheep were randomly selected and examined for presence of ticks from 2010 to 2011. Seven species of ixodid ticks were identified including *R. sanguineus, R. decoloratus, R. evertsi, H. marginatum, A. variegatum, R. annulatus,* and *H. sulcata* [147]. Nineteen species of ticks have been reported from Yemen including *A. variegatum, R. annulatus, R. kohlsi, H. excavatum, H. dromedarii, H. impeltatum, H. anatolicum, H. arabica, H. truncatum, H. rufipes, R. evertsi, R. sanguineus, R. simus, R. decoloratus, H. marginatum, H. sulcata, R. camicasi, R. turanicus* and *O. savignyi* (Table 1).

### 5.3. Common Ticks of the MENA Region

**Figure 2:**
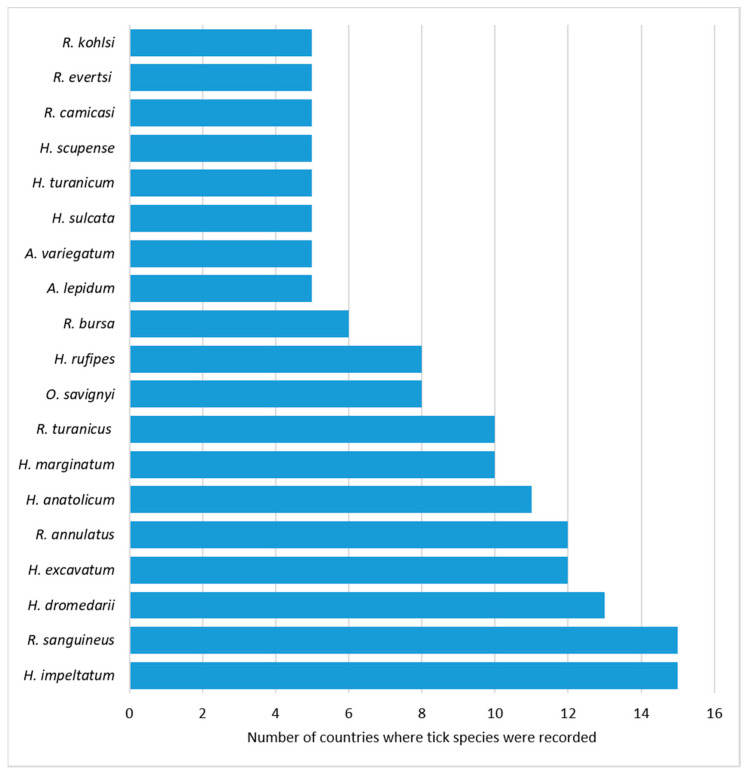
Tick species reported in more than four countries in the MENA (Middle East and North Africa) region.

Several tick species have common and widespread distribution in the MENA region. For example, *H. impeltatum* and *R. sanguineus* were reported in 15 countries, followed by *H. dromedarii* that was in turn found in 13 countries (Figure 2) covering a wide geographical distribution. These findings show that countries which have common tick species need to conduct joint research projects especially amongst countries with share borders where tick cross-border movement is a very likely mechanism of disease spreading. In addition, when a tick species is reported in 15 countries, this raises concerns about tick-borne disease transmission between these countries through the movement of people or livestock. For example, malignant theileriosis could be vectored by *H. impeltatum* to sheep and goats in the 15 counties in which it is found (Table 1). Likewise, the same could be said about the probable spread of ehrlichiosis and babesiosis in the countries where *R. sanguineus* is found. Thus, international cross-border management strategies involving all countries must be in place to curb the threat of tick-borne diseases.

**Figure 3:**
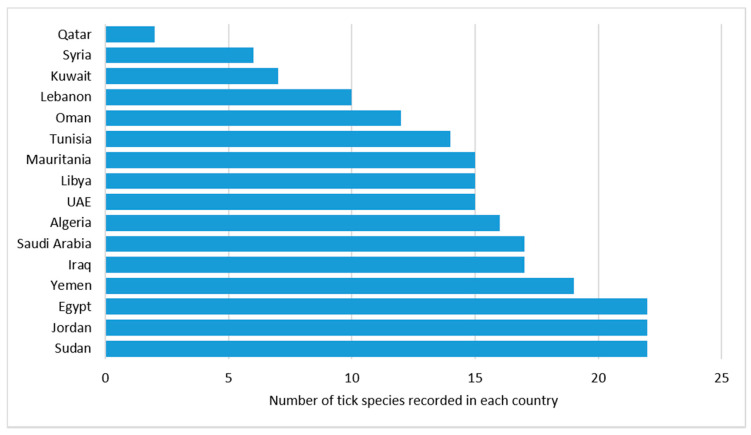
Arab countries with number of tick species reported. Sudan, Jordan, and Egypt have the largest number of reported ticks (N = 22) followed by Yemen, Iraq, and Saudi Arabia (N = 19, 17, 17), respectively.

This review paper shows that Sudan, Jordan, and Egypt has the largest number of reported tick species (N = 22) followed by Yemen, Iraq, and Saudi Arabia (N = 19, 17, 17), respectively (Figure 3). These findings have two interpretations. First, the high number of reported tick species in one country could be a result of conducting more research projects and the availability of entomologists in the country. Second, it is likely that there are more ticks in these countries, which calls for establishing effective tick management programs because they are at higher risk of tick-borne diseases compared to countries with less tick species. Moreover, countries such as Qatar, Syria, and Kuwait need to check if they have enough tick surveys in place to make sure that the low numbers are not the result of inadequate screening. The same applies to the other countries with lower tick numbers.

## 6. Tick-Borne Diseases

Vector-borne zoonotic diseases have emerged or re-emerged in many geographical regions causing global health and economic problems that involve humans, pathogens, vectors and wildlife [148,149]. Many tick-borne diseases such as viral, bacterial, and protozoan have been reported in the Arabian Peninsula. Ticks can be infected with bacteria, viruses or protozoa [23,150,151,152,153,154]. Co-infection of multiple tick-borne pathogens is usually found in dogs [155,156,157]. Transmission and persistence of vector-borne diseases depend on the overlapping distributions of hosts and vectors combined with the correct set of environmental conditions for a given pathogen [4,158]. All these tick-transmitted diseases (including babesiosis, theileriosis, anaplasmosis and cowdriosis) cause important economic losses to the livestock industry, mainly affecting tropical and subtropical countries, where ticks constitute one of the main difficulties for the development of the livestock breeding industry [23,159].

From the medical and veterinarian perspective, most of arthropod-borne infections can be associated with 116 tick species (32 argasid species and 84 ixodids). In addition, tick-borne diseases are common in the medical and veterinary clinical settings and a good surveillance is essential for the management of zoonosis and communicable diseases that are common to humans and animals [160].

**Figure 4:**
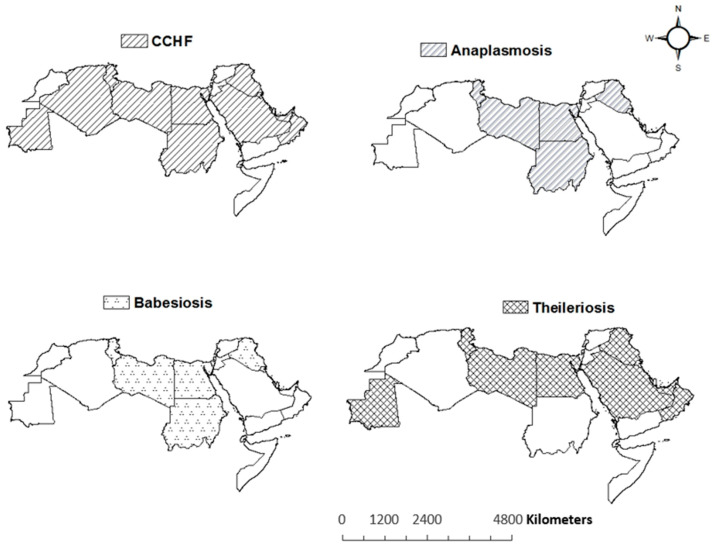
The geographic distribution of tick-borne diseases (CCHF, Anaplasmosis, Babesiosis and Theileriosis) in Arab countries.

In the Arab countries, the ticks that transmit diseases belong to both the soft-tick family, Argasidae (genera *Argas*, *Ornithodoros* and *Otobius*), as well as the hard-tick family, Ixodidae (genera *Dermacentor, Hyalomma, Haemaphysalis, Ixodes, Amblyomma* and *Rhipicephalus*) [41,43,63,98,161,162]. The main tick-borne diseases of veterinary importance in the tropical countries are theileriosis, babesiosis, cowdriosis and anaplasmosis [66]. However, we documented in this review following major tick-borne diseases of human and animal importance in Arab counties: protozoa diseases (babesiosis and theileriosis), bacterial diseases (anaplasmosis, ehrlichiosis, Lyme borreliosis, Mediterranean spotted fever, spotted fever *Rickettsioses*, tick-borne relapsing fever and Tularemia), and viral diseases (Alkhurma hemorrhagic fever, Crimean–Congo hemorrhagic fever, and tick-borne encephalitis) (Table 2). The distributions of four widely occurring tick-borne diseases in the MENA region are illustrated in Figure 4.

### 6.1. Viral Diseases

#### 6.1.1. Alkhurma Hemorrhagic Fever Virus (ALKV)

This virus is a recently described member of the tick-borne hemorrhagic fever group of the genus *Flavivirus*. Camels and sheep are thought to be the natural hosts of ALKV [202]. ALKV was recently detected in an *O. savignyi* tick collected near Jeddah, Saudi Arabia [200]. In the Arabian Peninsula, these ticks have been associated with camels and their resting places and can be found where cases of ALKV infection in humans have been reported. Overall, Alkhurma virus was reported from Egypt [202] and Saudi Arabia [197,198,203,204,240] (Table 2).

#### 6.1.2. Crimean–Congo Hemorrhagic Fever (CCHF)

CCHF has been reported from more than 30 countries in Africa, Asia, South-East Europe, and the Middle East. The majority of human cases are workers in livestock industry, agriculture, slaughterhouses, and veterinary practice [241,242]. The infection of CCHF is enzootic, however, mostly asymptomatic in many animal species such as cattle, sheep, goats, and camels [173,243]. Thirty species of ticks, particularly the genus *Hyalomma*, act as both reservoir and vector for this pathogen. Humans become infected by tick bites or through close contact with infected animals and humans [242]. *Hyalomma* ticks were responsible for outbreaks in humans with high fatalities in the UAE, Oman, and Saudi Arabia [165,172]. CCHF virus survives trans-stadially (from larva to nymph to adult) and inter-seasonally in several tick species and is transmitted transovarially to the F1 generation (in some cases to F2) in *H. marginatum*, *H. rufipes*, *D. marginatus* and *Rhipicephalus rossicus* [164]. CCHF disease causing virus has been reported from almost all countries of Arabian Peninsula (Table 2) [163,166,172,174,176,180,182,183,184,188,189,193].

#### 6.1.3. Tick-Borne Encephalitis (TBE)

This vector-borne disease is caused by the tick-borne encephalitis virus (TBEV) which belongs to *Flavivirus* genus, and has been a growing public health concern in Europe and other parts of the world for the past 20 years [244]. Ticks are considered both vectors and reservoirs for TBEV [245], humans can also acquire TBE infections by consuming unpasteurized milk and cheese [246]. TBEV, the causative agent, is transmitted to humans through the bite of an infected vector tick (*I. ricinus* or *I. persulcatus*). The vertebrate hosts of *I. ricinus* and *I. persulcatus*, which may have TBEV, are wild and domestic animals. The virus prevalence in ticks can vary substantially within and among risk areas [244]. Various species of mammals and migratory birds also play an important role in the transmission and distribution of TBEV [247]. TBE cases have been reported from Saudi Arabia in 1995 [248]. To date, no causal treatment is known, however, infection and disease can be prevented by avoiding tick bites (using insect repellents, etc.) and vaccination [244]. Therefore, this could be emerging disease in Arab world or Middle East.

### 6.2. Bacterial Diseases

#### 6.2.1. Anaplasmosis

It occurs in tropical and subtropical areas throughout the world and the disease is a major constraint to cattle production in many countries [249]. Anaplasmosis is a widely spread tick-borne infection of humans and animals, caused by *Anaplasma* species including *A. marginale, A. centrale, A. bovis,* and *A. ovis* for ruminants, and *A. phagocytophilum* for human and domestic animals [250]. The disease in humans is called ‘Human Granulocytic Anaplasmosis’ [251] and in cattle is called bovine anaplasmosis [249]. Belkahia et al. [210] reported three *Anaplasma* species including *A. marginale, A. centrale,* and *A. bovis*, from Tunisian cattle. Whereas, Awad et al. [208] reported *A. marginale* in cattle in northern Sudan and *R. annulatus* was the vector. Anaplasmosis has also been reported from other countries including Libya, Egypt, Iraq and Palestine (Table 2) [75,212,213].

#### 6.2.2. Ehrlichiosis

This tick-borne disease has been detected from both humans and animals. In humans, ehrlichiosis is called ‘human monocytotropic ehrlichiosis’ and caused by *A. phagocytophilum, Ehrlichia chaffeesis,* and *Ehrlichia ewingii*. The diseases are transmitted by *ixodid* ticks [250]. Ehrlichiosis is reported from Egypt, Palestine and Sudan (Table 2) [213,214,215].

#### 6.2.3. Mediterranean Spotted Fever (MSF)

Mediterranean spotted fever is a tick-borne disease caused by *Rickettsia conorii*. It was first described a century ago as a disease associated with high fever and spots. Conor and Bruch [252] first described the disease in Tunisia in 1910, and it was soon reported in other regions around the Mediterranean basin. *R. conorii* is an obligate intracellular, Gram-negative bacterium that is extremely fastidious [252]. *R. sanguineus* is the main vector of the disease. Humans are an accidental host of *Rickettsia*, and they have no role in maintaining this bacterium in nature [217]. The bacterium can affect people of all ages. Mediterranean spotted fever has been reported from Arab countries including Algeria, Morocco, Sudan, Jordan, Mauritania and Tunisia (Table 2) [140,205,216,217].

#### 6.2.4. Spotted Fever *Rickettsioses* (SFR)

Spotted fever *Rickettsioses* is an important tick-borne disease group in animals and humans in the world. SFR includes rocky mountain spotted fever (RMSF), Pacific Coast tick fever, and Rickettsial pox. The Rickettsial pathogens are categorized into order Rickettsiales, which includes the family Rickettsiaceae and Anaplasmataceae [253,254]. Most of the rickettsial diseases are caused by infection with obligate intracellular Gram-negative bacteria transmitted by arthropod vectors [252,255]. Al-Deeb et al. [143] recorded spotted fever group *Rickettsia* sp. in *H. dromedarii* ticks in the UAE. In addition, Demoncheaux et al. [140] reported *Rickettsia aeschlimannii* in *H. dromedarii* ticks from Tunisia [140] and Loftis et al. [215] detected *R. aeschlimannii* in *Hyalomma* sp. from Egypt [215].

#### 6.2.5. Tick-Borne Lyme Borreliosis

This is a zoonotic disease transmitted by hard ticks of the genus *Ixodes* [256,257] and most common in temperate forested regions of North America, Europe and Asia [258]. Globally, Lyme disease is caused by some members of the *B. burgdorferi* sensu lato (s.l.) species complex including *B. burgdorferi* sensu stricto in North America [259] and five species in Europe, *Borrelia afzelii, B. garinii, B. burgdorferi, Borrelia spielmanii* and *Borrelia bavariensis* [260]. Nymphs of *I. scapularis* ticks transmit *B. burgdorferi* to humans far more frequently than adult ticks. Efficient transmission of this spirochete requires a minimum of 24 to 48 h of tick attachment, at which time the nymph obviously is engorged with blood [261]. There are tens of thousands of clinical cases of Lyme disease reported annually. However, with the estimation of underreporting, there are hundreds of thousands of infections each year [258]. Developing novel control methods and predicting disease risk to better target control interventions require understanding pathogen and disease dynamics [258]. Various pathogens such as *B. burgdorferi, B. lusitaniae, B. garinii* were reported in *I. ricinus* from Tunisia [137]. However, *B. burgdorferi* was detected in ixodid ticks (*R. annulatus*, *H. dromedarii*, *H. excavatum* and *R. sanguineus*) and soft ticks (*O. savignyi*) from Egypt in 2010 and 2014 [219,220] and in *I. ricinus* from other Middle East and North African countries [162] (Table 2).

#### 6.2.6. Tick-Borne Relapsing Fever (TBRF)

Relapsing fever cases were reported from Sudan in 1939 [221], from Egypt in 1954 [37] and from Jordan in 1957 in humans [98]. TBRF is attributed mainly to spirochete *Borrelia*. *Ornithodoros* is the most important tick vector, found in Iraq, Syria, Jordan, and Egypt [223]. However, a louse-borne relapsing fever outbreak in Sudan was estimated to have affected 20,000 members of the Dinka tribe in 1998 and 1999; the death rate was 10–14% [262]. Reports of tick-borne relapsing fever appear to be involved in clusters of infection, associated with exposure of susceptible human hosts to tick vectors and domestic animals. In the Middle East, *B. persica* infections were transmitted by *O. tholozani* vector, in 2005 [262].

#### 6.2.7. Tularemia

This is a zoonotic disease that is caused by the gram-negative bacterium *F. tularensis* [263,264]. Ticks play important role in the epidemiology of Tularemia as a reservoir and vector, and can carry the bacterium by both of trans-ovarial and trans-stadial transmission [226]. Amblyoma, Dermocentor and Ixodes species are considered the main vector responsible for transmission [226]. In Arab countries, recently genus Francisella is reported in *H. dromedarii* from Egypt [227] and UAE [228]. Further, the presence of *F. tularensis* IgG antibodies in some patients in Egypt [227] poses a serious threat about emergence and re-emergence of Tularemia due to high prevalence of tick species in Arab world [142,144].

### 6.3. Protozoal Diseases

#### 6.3.1. Babesiosis

This is a zoonotic infection of animals and humans, caused by *Babesia* spp., and transovarially and trans-stadially transmitted by Ixodid ticks [265]. The disease is characterized by high temperature, anemia, restlessness, anorexia, and death. Babesiosis is seen in domestic animals, including sheep and goats, however the major economic impact of this infection is on the livestock industry worldwide due to high losses [231,250,266]. Babesiosis was investigated in blood samples of sheep and goats in Kurdistan-Iraq from June to September 2012 and four *Babesia* species were detected: *B. ovis, B. motasi, B. foliata,* and *B. taylori*. One species of tick vector *H. anatolicum* was found on these animals [231]. *Babesia* spp. have also been reported from Egypt, Libya, Tunisia, Iraq, Sudan, Saudi Arabia [61,75,207,208,229,230] (Table 2).

#### 6.3.2. Theileriosis

This is one of the most common tick-borne diseases, which have been studied in a wide range of livestock such as cattle, sheep, and goats. Few people studied theileriosis infected camels [41,43,267]. Theileriosis is caused by *Theileria* (obligate intracellular protozoan parasites) transmitted by ixodid ticks (*Hyalomma* spp.), which yields severe and mild infections in their vertebrate hosts [43]. This pathogen (*Theileria* spp.) has been described in all livestock species and can cause significant economic losses to farmers [268]. *Theileria* sp. has been reported from most of countries in Arab region including Egypt, Sudan, UAE, Mauritania, Saudi Arabia, Oman, Libya, Tunisia and Iraq [43,75,121,141,143,207,229,238] (Table 2).

## 7. Ticks and Livestock Industry

Ticks are presented with a unique set of challenges and opportunities in the Arab region. The natural environment is arid and inhospitable, with pockets of habitat that periodically become productive [24]. This is a challenge for the survival of hosts and therefore also for ticks. However, the widespread occurrence of livestock in the region, presents ticks with the opportunity to thrive in high densities in artificially improved conditions (e.g., with shelter and water provided for animals). Although there have been efforts to modernize the livestock industry, local farms with low to medium density of livestock abound in the region. These farms have frequent input of imported livestock since cattle, sheep and goats are regularly imported into the MENA region from Australia, New Zealand, China and Argentina [269]. Local populations of ticks, therefore, have opportunities to feed on naïve hosts from outside the region, with substantial chances of boosting populations to levels much higher than what could be normally supported in an arid environment. Thus, ticks have been enhanced in the region and farms represent areas of high population density and evolution of ticks. Specifically, the use of acaricides is widespread and without any form of vector control [270]. Thus, ticks have the continuous opportunity to develop resistance to acaricides [270,271]. Frequent influx of foreign host animals also raises the chance of importing pathogens, especially those that may remain dormant in the host [272]. This is discussed further in the following section.

## 8. Infectious Diseases and Global Movement of Humans and Domestic Animals

With the development of effective vaccines, antibiotics and improved sanitation, infectious diseases were significantly reduced in the developed world in 1970s. However, the emergence of a series of new diseases and global spread of HIV/AIDS, led to infectious diseases being considered with increasing priority in health policy and political agendas [273,274]. More than 60% of human infectious diseases emerging between 1940 and 2004 were zoonotic, resulting in significant global morbidity, mortality, and economic costs [275]. Of those emerging zoonoses, 71.8% are from wildlife and 22.8% are arthropod vector-borne infections [275]. Significantly, the frequency of emerging vector-borne zoonoses has increased during the last ten years [275,276]. Public concern about emerging infectious diseases was increased because of their often rapid spread and high case fatality rates [277,278]. Infectious diseases emergence was associated with environmental or human behavioral change and human interaction with wildlife [279]. Emergence was found to be intensified by increasing human travel and globalized trade [280]. New infectious diseases continued to emerge, often from unexpected reservoirs and via new pathways [4].

Travel, trade and an altered attitude towards domestic animals, wildlife and nature influence vectors and pathogens distribution worldwide. The Middle East imports mostly goats, sheep and cattle from as far as Australia, China and Argentina, often using mixed transportation systems [269]. Mixed transportation often results in considerable morbidity and mortality of livestock. However, surviving livestock can serve as a source for non-native tick species that could help to import non-native pathogens. Furthermore, increased tick densities could permit non-native pathogens to enter and maintain circulation within farming systems. The fundamental problem and trigger for these effects, however, seems due to the increased human population size and associated farming activities [281]. There are multiple causes of novel disease emergence; however the human-mediated transport of pathogens or vectors across geographical or ecological boundaries (pathogen pollution) has been identified as a major driver of diseases emergence in animals [4]. Therefore, it is very likely that tick-borne diseases will remain within farming systems and will become an increasing problem [281].

There are some scattered reports in the literature of *Hyalomma* species being imported into the USA, most-commonly on animals and animal products. Mertins and Schlater [282] documented five species of *Hyalomma* on ostriches imported from Africa and Europe. However, Keirans and Durden [283] reported one case of *H. marginatum* found on a human with travel history to Greece. A detailed travel history may be important for identification of ticks, as well as to assess the risk of vector-borne diseases.

*Hyalomma* is one of the most medically-important tick genera in the region. Species in this genus have been reported to transmit a variety of viral, bacterial, and parasitic diseases of medical and veterinary importance [27]. One of the most crucial human viruses transmitted by *Hyalomma* spp. is CCHF virus. Sexually and transovarial transmission of CCHF virus was observed experimentally in *H. truncatum* [284]. In fact, comprehensive documents and research papers exist showing that several tick species transmit the bacteria, virus and parasites in different geographical areas of the world [285]. For instance, *H. marginatum* transmitted the CCHF in Russia, Turkey, and Crimean Peninsulas, *H. anatolicum* in Iran, Pakistan, Turkmenistan, and Tajikistan, *H. asiaticum* from Central Asia to China, and *H. rufipes* in Africa [27,286,287]. Other important disease agents of humans and their documented vectors include *R. conorii* in *H. rufipes* and *H. truncatum*, *R. aeschlimannii* in *H. marginatum, H. truncatum*, and *H. scupense*, *R. sibirica* in *H. asiaticum, H. excavatum*, and *H. truncatum*, *A. phagocytophilum* in *H. lusitanicum*, and *C. burnetii* in *H. scupense* [27,287,288]. *Hyalomma* species have also been implicated in tick paralysis in humans [289].

Thousands of livestock are imported annually from Arabian countries such as Sudan, Somalia and others such as Turkey, Argentina, Pakistan, Australia Iran and Uruguay in Saudi Arabia and UAE. Camel, sheep, goat and cattle production plays an integral part in the agricultural sector and contribute significantly to the food security of the country [11]. Prior to 1995, tick records from the UAE were limited. Records on file at the U.S. National Tick Museum indicate that only the ixodid ticks, *H. anatolicum*, *H. impeltatum, H. dromedarii, R. sanguineus,* and *R. turanicus*, were reported in the UAE before CCHF outbreak in UAE 1994–1995 [39]. Ticks collected during this CCHF outbreak investigation represent the largest group of ticks collected to date from the UAE and indicate that several competent vectors of CCHF virus were being imported [39]. Results of this investigation indicated that most CCHF virus-infected animals were imported from Somalia, with fewer numbers of infected animals possibly arriving from Iran [39].

Little efforts have been made to put in place the policies to reduce risk. Determining and preventing the importation of infected hosts is widely used to stop importation of many domestic animal diseases of economic or public health importance. Some countries even endorse this principle for the movement of people, whereby they conduct surveillance for infected persons arriving at their international borders, particularly during human pandemics. The World Health Organization (WHO) provides guidance and training through its international health regulations (IHR). Rules and regulations for international trade including animals and their products are created and enforced by the WHO, for smooth flow of trade [4].

## 9. Climate Change and Ticks and Tick-Borne Diseases

Like the rest of the world, Arab countries are affected by climate change. Climate change is considered one of the many factors that play a role in tick abundance and distribution. Climate change impact (positive or negative) on the biology of ticks (e.g., higher mean temperatures and increased humidity facilitate tick survival on certain latitudes, in other regions the opposite effect occurs). Other factors such as habitat fragmentations, demographic modifications and other environmental changes may also be involved and complicating the processes. These factors may also facilitate the survival and establishment of colonies in regions where tick species were not prevalent before [290]. The knowledge regarding the disease causing pathogens and their epidemiology have significantly improved during the last decade, further studies are required to explain the lifecycle of these pathogens, (viruses, bacteria and protozoa) antigenic differences and human immune response for effective treatment and vaccine development [281]. Developing novel control methods and predicting disease risk to better target control interventions require understanding of pathogen and disease dynamics [258]. Meanwhile, the best way for prevention of the disease is the education of risk groups (especially farm labors and workers at slaughter houses/abattoir) and awareness in healthcare workers.

The distribution and abundance of tick populations also depend on the interaction of large-scale climate influences, local scale microclimates, habitat characteristics (including tick predators) and host densities [258]. Microclimate conditions may impact tick survival directly by increasing tick mortality rates, and indirectly by influencing the activity [291,292]. Both cold and hot temperatures significantly decrease the tick survival and host-seeking activity. Low humidity reduces activity and can kill ticks quickly through desiccation; high levels of rainfall also inhibit activity [293]. In adverse climate conditions, ticks often seek microclimate refuges in leaf litter or debris to reduce the impacts of extreme temperature and humidity on survival [294]. The risk of human infection thus is thought to increase with the density of questing infected nymphs in the environment, which is the product of the density of nymphs and their infection prevalence, and varies at a very local scale [258].

## 10. Strategies for Prevention and Control of Infectious Diseases

One health approach is used to tackle the zoonotic diseases by considering all components including environmental and ecological/wildlife as well as domestic animal and human factors (cultural, political and other socio-economic drivers) [1]. The success of this multi-disciplinary approach has been driven by combining the field sciences with analytical approaches and laboratory science. Challenges remain, however. Disease control or prevention is best achieved through integrated public health, veterinary medicine, animal management and ecological approaches. Health approaches are also mandatory at the policy and governance levels and these become successful and cost-effective if developed and implemented by all relevant parties including ecologists, conservation biologists, policy-makers and experts from veterinary and medical professions [4].

## 11. General Recommendations

### 11.1. Lack of Publication Record in Arab Countries

We found no published research papers on ticks and tick-borne pathogens in some Arab countries, despite the presence of animals in them. This could be interpreted in two ways. One possibility is that there might be no tick infestations in the country, which is very unlikely, knowing how prevalent the ticks are in the region. The second possibility might be that there is no research done on ticks, which could be the result of having different problems that need to be investigated and fixed. Whatever the case might be concerned authorities in each country should encourage tick-related research and provide necessary resources especially financial support.

### 11.2. Need of Mutual Collaboration

According to published papers, some tick species were common in neighboring countries that share joint borders. This calls for mutual collaboration among such countries to study and stop tick cross-border movement. In addition, the success of any tick control program in one country is always going to be reliant on good collaboration from the country on the other side of the border. Otherwise, it will serve as a tick reservoir from which ticks continue to cross into the border and reestablish infestations.

In order to enhance tick control efforts, inter-country research projects should be established and supported by inter-country funding. This is very important for the management of tick species that are common in more than one country. Moreover, establishing a central collection of tick specimens and a repository for DNA and RNA samples extracted from different tick species in each country can facilitate and enhance the research on ticks. Consequently, tick management will be more successful over time. In addition, the collaboration among research teams in different countries will be more successful and effective.

### 11.3. Need to Create Awareness through Workshops and Conferences

It is important to organize awareness creation workshops to ensure that the reporting of ticks and tick-borne pathogens is going to be a routine practice for people dealing with animals and that this is always going to be done in a proper and timely manner. This is because some animal care providers do not fully appreciate the importance of publishing research results or reporting the presence of tick and tick-borne pathogens as long as proper medicine and treatment are given to affected animals.

### 11.4. Animal Trade (Import and Export) Regulations

Animal trade (import and export) regulations concerning border inspections of animals for the presence of ticks and tick-borne pathogens should be standardized among all Arab countries. This practice can eliminate, or at least minimize, any infiltration of ticks into a new country as a result of lenient inspection on some points of entry.

### 11.5. Tick Surveillances Programs

There is a need for conducting comprehensive tick surveillances (qualitative and quantitative) in each Arab country in order to know tick species and hosts. The results should be coupled with tick species mapping to determine the geographical distribution of tick infestations and the hot spots in each country. Latest mapping software and global positioning system data should be utilized.

### 11.6. Reference Laboratory

It is vital to establish a standard reference laboratory in each Arab country to identify tick-borne pathogens and to serve as an information resource and point of contact at the national, regional, and international levels.

### 11.7. Cutting-Edge Research Protocols

There is a need to encourage the use of cutting-edge research protocols for studying and identifying ticks and tick-borne pathogens with an emphasis on the use of molecular tools and next generation sequencing.

## 12. Conclusions

Because the ixodid tick species reported in this review infests both animals and humans, they have veterinary and medical importance. The increase in tick-borne diseases has been attributed to a range of factors that include habitat fragmentation, changes in host communities, human travel and trade, and climate change. The ending of critical gaps in tick-borne diseases ecology research would significantly improve our ability to forecast the location and timing of hot spots of these infectious diseases and to target control efforts at the most important phase of the transmission cycle. Efficient prevention and control require the understanding of the local ecology and human behavior.

## Figures and Tables

**Table 1:** Tick species distribution in Arab countries.

Families	Genera	Species	Arab Countries	Hosts	References
Argasidae	*Ornithodoros*	*O. erraticus*	Iraq, Jordan, Saudi Arabia	Animals	[28,29]
		*O. foleyi*	Oman, Libya	Farm animals	[29,30]
		*O. lahorensis*	Jordan		[28]
		*O. salahi*	Egypt, Jordan		[28,31]
		*O. savignyi*	Algeria, Egypt, Lebanon, Libya, Mauritania, Oman, Saudi Arabia, Yemen	Camels	[29,32,33,34,35,36]
		*O. tholozani*	Egypt, Libya, Jordan	Humans	[28,35,37]
	*Otobius*	*O. megnini*	Iraq	Buffaloes	[38]
Ixodidae	*Amblyomma*	*A. gemma*	Saudi Arabia, UAE, Yemen	Cows, camels	[29,39,40]
		*A. lepidum*	Egypt, Iraq, Saudi Arabia, Sudan, UAE	Livestock, camels	[29,39,41,42]
		*A. marmoreum*	Egypt	Camels	[41]
		*A. variegatum*	Egypt, Oman, Saudi Arabia, Sudan, Yemen	Camels	[29,33,41,43]
		*A. latum*	Saudi Arabia, Yemen	Domestic animals	[29,40]
	*Dermacentor*	*D. marginatus*	Algeria, Lebanon	Cattle	[44,45]
	*Haemaphysalis*	*H. erinacei*	Algeria, Iraq, Jordan	Animals	[28,46]
		*H. indica*	Oman		[29]
		*H. parva*	Iraq, Libya, Jordan	Domestic animals	[28,35,47,48]
		*H. punctata*	Algeria, Lebanon, Tunisia	Domestic animals	[45,49,50]
		*H. sulcata*	Iraq, Jordan, Saudi Arabia, Tunisia, Yemen	Sheep	[28,33,46]
	*Hyalomma*	*H. anatolicum*	Egypt, Iraq, Jordan, Kuwait, Lebanon, Oman, Saudi Arabia, Sudan, Syria, UAE, Yemen	Livestock	[40,46,47,51,52]
		*H. arabica*	Yemen	Goats, sheep	[33]
		*H. asiaticum*	Iraq	Cattle, sheep	[52]
		*H. dromedarii*	Algeria, Egypt, Iraq, Jordan, Kuwait, Mauritania, Oman, Qatar, Saudi Arabia, Sudan, Tunisia, UAE, Yemen	Camels	[33,41,43,53,54,55]
		*H. excavatum*	Algeria, Egypt, Iraq, Jordan, Lebanon, Libya, Saudi Arabia, Sudan, Syria, Tunisia, UAE, Yemen	Cattle, camels	[33,41,52,54,56]
		*H. franchinii*	Libya, Tunisia	Camels, sheep	[30,50]
		*H. hussaini*	UAE	Livestock	[39]
		*H. impeltatum*	Algeria, Egypt, Iraq, Jordan, Kuwait, Libya, Mauritania, Oman, Qatar, Saudi Arabia, Sudan, Syria, Tunisia, UAE, Yemen	Camels, cattle, goats	[39,41,52,53,54,57,58]
		*H. impressum*	Algeria, Mauritania, Sudan	Camels	[53,57]
		*H. lusitanicum*	Algeria	Cattle	[44,49]
		*H. marginatum*	Algeria, Egypt, Iraq, Jordan, Kuwait, Libya, Syria, Tunisia, UAE, Yemen	Camels, cattle, sheep	[47,52,54,56,59]
		*H. nitidum*	Mauritania		[36]
		*H. rufipes*	Algeria, Egypt, Libya, Mauritania, Oman, Saudi Arabia, Sudan, Yemen	Camels, cows	[41,53]
		*H. scupense*	Algeria, Iraq, Jordan, Sudan, Tunisia	Cattle, sheep	[28,46,52,56,59]
		*H. schulzei*	Jordan, Kuwait, Lebanon, Saudi Arabia	Domestic animals	[11,28,48]
		*H. truncatum*	Egypt, Mauritania, Sudan, UAE	Livestock, camels	[39,41,57]
		*H. turanicum*	Egypt, Iraq, Jordan, Libya, Saudi Arabia	Sheep, goats	[46,48,52]
	*Ixodes*	*I. hoogstraali*	Oman		[29]
		*I. ricinus*	Algeria, Tunisia	Cattle, sheep	[50,56,60]
		*Ixodes* sp.	Jordan	Animals	[28]
	*Rhipicephalus*	*R. annulatus*	Algeria, Egypt, Iraq, Jordan, Kuwait, Lebanon, Libya, Oman, Sudan, Syria, Tunisia, Yemen	Cattle	[33,41,51,54,61,62]
		*R. appendiculatus*	Sudan, UAE	Livestock, camels	[63]
		*R. bursa*	Algeria, Iraq, Jordan, Lebanon, Libya, Tunisia	Sheep, goats	[46,47,59]
		*R. camicasi*	Egypt, Jordan, Saudi Arabia, Sudan, Yemen	Sheep	[28,41]
		*R. decoloratus*	Libya, Sudan, Yemen	Cattle	[30,57,63,64]
		*R. evertsi*	Libya, Mauritania, Sudan, UAE, Yemen	Livestock, camels	[30,39,65]
		*R. geigyi*	Mauritania	Livestock	[57]
		*R. guilhoni*	Egypt, Mauritania, Sudan	Sheep	[41,57]
		*R. kohlsi*	Jordan, Iraq, Jordan, Saudi Arabia, Yemen	Goats, sheep	[33,46]
		*R. microplus*	Libya	Goats	[30]
		*R. muhsamae*	Sudan	Cattle	[66]
		*R. praetextatus*	Saudi Arabia, Sudan	Livestock	[67]
		*R. pulchellus*	Egypt, UAE	Camels, livestock	[43,68]
		*R. sanguineus*	Algeria, Egypt, Iraq, Jordan, Kuwait, Lebanon, Libya, Mauritania, Oman, Saudi Arabia, Sudan, Syria, Tunisia, UAE, Yemen	Livestock	[33,59,65,68]
		*R. simus*	Sudan, Yemen	Sheep, cattle, camels	[33,68,69]
		*R. sulcatus*	UAE	Livestock, camels	[39]
		*R. turanicus*	Algeria, Egypt, Iraq, Jordan, Lebanon, Oman, Saudi Arabia, Sudan, Tunisia, Yemen	Cattle, sheep, goats	[41,46,47,51,59]

**Table 2:** Tick-borne diseases, pathogens, vectors and hosts in Arab countries.

Pathogens	Host Species	Tick Vectors	Locality	References
CCHFV	Camels, sheep	*H. anatolicum, H. marginatum H. rufipes, H. impeltatum, R. sanguineus, R. turanicus, R. annulatus*	Egypt	[163]
CCHFV	Human, livestock		Egypt	[164]
CCHFV	Patients		United Arab Emirates	[165]
CCHFV	Sheep, cattle	*H.* *marginatum*	Iraq	[166]
CCHFV	Died patient		United Arab Emirates	[167]
CCHFV	Patients		Kuwait	[168]
CCHFV	Patients		Mauritania	[169]
CCHFV	Patients		Mauritania	[170]
CCHFV	Camel		Egypt	[171]
CCHFV	Livestock	*Hyalomma* sp.	Oman	[172]
CCHFV	Patients		United Arab Emirates	[173]
CCHFV	Camels, cattle, goat	*H. impeltatum H. excavatum H. anatolicum*	United Arab Emirates	[39]
CCHFV	Livestock	*Hyalomma* sp.	United Arab Emirates	[174]
CCHFV			Saudi Arabia	[175]
CCHFV	Domestic livestock	*H. anatolicum, R. evertsi*	Oman	[176]
CCHFV	Livestock	*A. variegatum, R. decoloratus, R. geigyi, H. impeltatum, H. impressum, H. rufipes, H. truncatum, R. guilhoni*	Mauritania	[65]
CCHFV	Patients		Sudan	[177]
CCHFV	Patients		Sudan	[178]
CCHFV	Livestock	*Hyalomma* sp.	Iraq	[179]
CCHFV	Patients		Oman	[180]
CCHFV	Patients		Sudan	[181]
CCHFV		*H. marginatum*	Iraq, UAE, Oman, Yemen, Saudi Arabia, Mauritania	[182]
CCHFV	Cow		Egypt	[183]
CCHFV	Cattle		Sudan	[184]
CCHFV	Cattle, camel, sheep, goat	*Hyalomma* sp.	Oman	[185]
CCHFV	Patients		United Arab Emirates	[186]
CCHFV	Cattle, camel, sheep		Oman	[185]
CCHFV	Patients		Mauritania	[187]
CCHFV	Patients		Tunisia	[188]
CCHFV			Algeria	[189]
CCHFV	Cattle		Mauritania	[190]
CCHFV	Camels		Sudan	[191]
CCHFV			Egypt	[192]
CCHFV	Humans		Iraq, Kuwait, Oman, Saudi Arabia, Sudan, UAE	[193]
CCHFV	Livestock		Egypt, Somalia, Tunisia	[193]
CCHFV	Patient		Oman	[194]
CCHFV	Cattle, camel	*H. rufipes, H. dromedarii, H. impeltatum*	Mauritania	[195]
CCHFV	Camel	*H. dromedarii*	United Arab Emirates	[196]
ALKV	Patients		Saudi Arabia	[197]
ALKV	Patients			[198]
ALKV	Patient, dead camel		Saudi Arabia	[197,199]
ALKV	Camels	*O. savignyi*	Saudi Arabia	[200]
ALKV	Camel, sheep	*H. dromedarii*	Arabian Peninsula	[201]
ALKV	Human		Egypt	[202]
ALKV	Human		Najran, Saudi Arabia	[203]
ALKV	Soldier		Jazan, Saudi Arabia	[204]
TBEV	Human, livestock	*I. ricinus*	Europe, North Africa, Middle East	[162]
*Rickettsia africae*	Humans	*O. foleyi*	Libya	[110]
*R. africae*	Humans		Mauritania	[205]
*R. africae*	Cattle	*A. variegatum, R. appendiculatus, R. microplus*	Comoros	[206]
*Anaplasma. marginale*	Cattle		Libya	[207]
*A. marginale*	Cattle	*R. annulatus*	Sudan	[208]
*A. marginale*	Cattle, sheep, goat	*R. sanguineus, R. turanicus, H. excavatum, H. anatolicum, H. marginatum, H. turanicum, H. scupense, R. annulatus*	Iraq	[209]
*A. ovis*	Sheep	*Rhipicephalus* sp.	Iraq	[75]
*A. marginale, A. central, A. bovis*	Cattle		Tunisia	[210]
*A. marginale*	Cattle		Libya	[211]
*A. marginale*	Buffalo		Egypt	[212]
*Anaplasma* sp., *Anaplasma platys*	Livestock	*Rhipicephalus* sp., *R. sanguineus*	Palestine	[213]
*Rickettsia conorii*		*R. sanguineus*	Libya	[112]
*Ehrlichia ruminatium*	Livestock	*A. variegatum, A. lepidum*	Sudan	[214]
*Ehrlichia* sp.	Livestock		Egypt	[215]
*Ehrlichia* sp.	Livestock	*R. sanguineus*	Palestine	[213]
*Rickettsia* sp.	Humans	*R.* *sanguineus*	Algeria, Morocco, Sudan	[216]
*R. conorii*	Humans		Mauritania	[205]
*R. conorii*	Camels	*H. dromedarii*	Tunisia	[140]
*R. conorii*	Humans	*R. sanguineus*	South Jordan	[217]
*R. aeschlimannii*	Livestock	*Hyalomma* sp.	Egypt	[215]
*R. aeschlimannii*	Cattle, sheep	*H. marginatum, H. scupense*	Algeria	[59]
*R. conorii, R. aeschlimannii*	Camels	*H. dromedarii*	Tunisia	[140]
*Rickettsia* sp.	Camels	*H. dromedarii*	UAE	[143]
*Coxiella burnetii*	Humans		Libya	[218]
*C. burnetii*	Humans		Mauritania	[205]
*Borrelia burgdorferi, B. lusitaniae, B. garinii*		*I. ricinus*	Tunisia	[137]
*B. burgdorferi*	Humans	*I. scapularis*	Egypt	[219]
*B. burgdorferi*	Humans		Egypt	[220]
*B. burgdorferi*	Human, livestock	*I. ricinus*	Europe, North Africa, Middle East	[162]
*Borrelia* sp.	Humans	*Ornithodoros* sp.	Sudan	[221]
	Humans	*O. tholozani*	Libya	[111]
*Borrelia* sp.	Humans	*Ornithodoros* sp.	Egypt	[37]
*Borrelia* sp.	Humans	*O. tholozani*	Jordan	[98]
*Borrelia persica*	Livestock	*O. tholozani*	Syria, Egypt	[222]
*Borrelia* sp.		*Ornithodoros* sp.	Iraq, Syria, Jordan, Egypt	[223]
*Francisella tularensis*	Human		Egypt	[224]
*F. tularensis*	Human		Iraq, Syria	[225]
*F. tularensis*	Human		Syria, Egypt, Lebanon	[226]
*Francisella* spp.	Camel	*H. dromedarii*	Egypt	[227]
*Francisella* sp.	Camel	*H. dromedarii*	United Arab Emirates	[228]
*Babesia bigemmina*, *B. ovis*	Camel, cows, sheep	*Hyalomma* sp., *Boophilus* sp., *Rhipicephalus* sp., *Amblyomma* sp., *Argas* sp.	Egypt	[41]
*B.bigemina*	Cattle		Libya	[207]
*B. bovis* *, B. bigemina*	Cattle	*R. annulatus* *, I. ricinus, H. punctata, H. sulcata, H.excavatum, H. scupense, H. marginatum*	Tunisia	[141]
*Babesia spp.*	Livestock	*H. scupense, R. bursa*	Tunisia	[139]
*B. bigemina, B. bovis*	Cattle	*R. annulatus*	Sudan	[208]
*B. occultans*		*H. marginatum*	Tunisia	[136]
*Babesia* sp.	Cattle	*H. anatolicum*	Iraq	[229]
*B. bigemina*	Cattle, sheep, goat	*R. sanguineus, R. turanicus, H. excavatum, H. anatolicum, H. marginatum, H. turanicum, H. scupense, R. annulatus*	Iraq	[209]
*B. ovis*	Sheep	*Rhipicephalus* sp.	Iraq	[75]
*Babesia* sp.	Camel		Saudi Arabia	[230]
*B. ovis, B. motasi, B. foliate B.taylori*	Sheep, goat	*H. anatolicum*	Iraq	[231]
*Babesia* sp.	Camel, cow	*O. savignyi, R. annulatus*	Egypt	[61]
*Theileria annulata*	Calf	*H. anatolicum*	Bahrain	[232]
*T. annulata*	Cow	*H. dromedarii*	Mauritania	[233]
*T. annulata, T. ovis, T. hirci*	Camel, Sheep, goat	*Ixodid* sp.	Saudi Arabia	[121]
*T. annulata*		*H. dromedarii*	Mauritania	[234]
*T. annulata*	Camels, cows, sheep	*Hyalomma* sp., *Rhipicephalus* sp., *Amblyomma* sp., *Argas* sp.	Egypt	[41]
*T. lestoquardi*	Sheep	*H. anatolicum*	Oman	[235]
*T. mutans*	Cattle		Libya	[207]
*T. annulata, T. buffeli*	Cattle	*R. annulatus* *, I. ricinus* *, H. scupense* *, H. sulcata, H. punctata* *, H.excavatum, H. marginatum*	Tunisia	[141]
*T. annulata*	Cattle	*Hyalomma* sp.	Iraq	[236]
*Theileria* spp.	Livestock	*H. scupense, R. bursa*	Tunisia	[139]
*T. lestoquardi*	Sheep		Oman	[237]
*Theileria* sp.	Cattle	*H. anatolicum*	Iraq	[229]
*T. ovis, T. lestoquardi, T. uilenbergi*	Sheep	*Rhipicephalus* sp.	Iraq	[75]
*T. ovis, T. annulata, T. lestoquardi*	Sheep	*Ixodes* sp.	Iraq	[161]
*T. annulata*	Camel	*H. dromedarii*	Egypt	[43]
*T. annulata*	Camel	*H. dromedarii*	United Arab Emirates	[143]
*T. annulata*	Cattle		Oman	[238]
*T. annulata, T. ovis, T. lestoquardi*	Cattle, sheep, goat	*H. anatolicum*	Oman	[239]
*T. annulata*	Camel	*H. dromedarii*	Egypt	[43]

CCHFV = Crimean–Congo hemorrhagic fever virus, ALKV = Alkhumra virus, TBEV = tick-borne encephalitis virus.
